# NAT10 mediated mRNA acetylation modification patterns associated with colon cancer progression and microsatellite status

**DOI:** 10.1080/15592294.2023.2188667

**Published:** 2023-03-12

**Authors:** Hailin Zhang, Wenqing Shan, Zhenwei Yang, Yangyang Zhang, Meng Wang, Liping Gao, Lingxiu Zeng, Qiu Zhao, Jing Liu

**Affiliations:** a Department of Gastroenterology, Zhongnan Hospital of Wuhan University, Wuhan, Hubei, China; b Hubei Clinical Center and Key Lab of Intestinal and Colorectal Diseases, Wuhan University, Wuhan, Hubei, China

**Keywords:** mRNA acetylation, NAT10, microsatellite status, colorectal cancer, tumor immune infiltration

## Abstract

N4-acetylcytidine (ac4C) is one type of RNA modification found in eukaryotes. RNA acetylation modifications are gradually expanding in oncology. However, the role of RNA acetylation modifications in colorectal cancer and its association with colorectal cancer microsatellite status remain unclear. Using public databases and in vitro experiments, we verified the expression and biological function of NAT10, as the key RNA acetylation modification enzyme, in colorectal cancer. The results showed that NAT10 was highly expressed in colorectal cancer, and significantly promoted colorectal cancer cell proliferation. NAT10 was also involved in several aspects of cell homoeostasis such as ion transport, calcium-dependent phospholipid binding, and RNA stability. NAT10 expression positively correlated with immune infiltration in colorectal cancer. We further constructed a risk regression model for mRNA acetylation in colorectal cancer using acetylation-related differential genes. We found that tumour immune infiltration, microsatellite instability (MSI) proportion, tumour immune mutation burden, and patient response to immunotherapy were positively correlated with risk scores. For the first time, our study showed that the level of mRNA acetylation modification level is elevated in colorectal cancer and positively correlates with immune infiltration and microsatellite status of patients. Based on our findings, NAT10 may be a new target for colorectal cancer treatment.

## Introduction

Colorectal cancer is the fifth leading cause of cancer-related in morbidity and the third leading cause of mortality globally [1]. As of 2020, the number of colorectal cancer cases has exceeded 1.9 million, and this number is expected to reach 3.2 million by 2040 [2]. Currently, the treatment of colorectal cancer mainly relies on traditional surgery, radiation therapy, and chemotherapy [3]. Recently, immunotherapy has gradually become a promising therapy for colorectal cancer patients. However, since the FDA approval of pembrolizumab, a programmed death-ligand 1 (PD-L1) monoclonal antibody, for colorectal cancer, only a subset of patients has effectively responded to immune-checkpoint inhibitors (ICIs) [4]. Furthermore, the specific mechanisms leading to immunotherapy resistance in colorectal cancer remains to be uncovered.

Colorectal cancer can be classified into microsatellite stable (MSS) and microsatellite instable (MSI) subtypes, based on mutations or DNA methylation of genes in mismatch repair (MMR) machinery [5]. Due to DNA mismatch repair deficiency, MSI Colorectal cancer typically contains numerous tumour neoantigens, which can induce immune cell infiltration and activate the immune response. In contrast, MSS colorectal cancer is insensitive to ICIs and evades immune infiltration due to DNA mismatch repair protein proficiency [5–7]. MSI colorectal cancer comprises only 15% of recurrent disease cases [8]. Therefore, it is of great importance to screen and develop new biomarkers with the potential to reverse the immune infiltration status of patients with MSS colorectal cancer and transform ‘cold’ tumours into ‘hot’ tumours.

Recently, the role of epigenetics and epigenomics in cancer has gradually been elucidated. RNA acetylation modification is a highly conserved RNA modification process that was first thought to exist in ribosomal RNA (rRNA) and transfer RNA (tRNA) [9]. However, recent studies have shown that acetylation modification can also occur in mRNAs, which is critical for maintaining the structural stability of mRNA [10]. N-acetyltransferase 10 (NAT10) is the only molecule catalysing the acetylation modification of human RNA. It plays an important role in the development of cancer. Previous studies have reported that NAT10 is closely correlated with disease progression in a variety of human tumours, including stomach cancer [11], prostate cancer [12], and bladder cancer [13]. However, the role of mRNA acetylation modification levels and the *NAT10* gene in colorectal cancer and their effect on immunotherapy were not investigated.

Using bioinformatics algorithms and in vitro experiments, we found that NAT10 promotes the development of colorectal cancer. Subsequently, we divided the colon cancer samples into two groups, namely the high NAT10 expression group and the low NAT10 expression group. Then, we identified 106 genes related to NAT10 by intersecting the differential genes of tumour and normal samples between the high NAT10 expression group and the low NAT10 expression group. Based on ac4Cscore, a risk prediction model was established using LASSO regression models. We then divided the patients into high-risk and low-risk groups based on ac4Cscore and further analysed their differences in prognosis, tumour microenvironment, and microsatellite status. Therefore, these results suggested that NAT10 and mRNA acetylation modification are involved in pathogenesis of colorectal cancer and can be potential targets for therapy.

## Methods

### Data retrieval and pre-processing

Gene expression data of colorectal cancer patients were obtained from The Cancer Genome Atlas (TCGA) (TCGA-COAD). The data were normalized using log2(count+1). Colorectal cancer cell line data were downloaded from the CCLE database. Immunohistochemical images of NAT10 expression in colon cancer tissues and normal colon tissues were obtained from The Human Protein Atlas.

### Functional enrichment analysis

Gene Ontology (GO) was used to annotate genes with functions, including molecular function (MF), biological pathways (BP), and cellular components (CC). Kyoto Encyclopedia of Genes and Genomes (KEGG) Enrichment Analysis was used as a practical resource for the analytical study of gene functions and associated high-level genome functional information. The R package ‘cluster Profiler’ was used for GO analysis and KEGG analysis. P-value Filter<0.05 and q-value Filter<0.05 were considered as statistically significant.

### Estimation of TME cell infiltration

To assess the immune status of the tumours, we used CIBERSORT and ESTIMATE algorithms to evaluate immune and stromal cells infiltration. The immunocyte population fractions were analysed by CIBERSORT R script v1.03. Stromal Score. Immune Score and Estimate Score were calculated by R software package ‘estimate.’

### Identification of differentially expressed ac4c-modified genes in colon cancer

We analysed the differential expression of genes between normal and tumour tissue named C1. Then, the Colon cancer patients were divided into high- or low-expression groups according to the median level of NAT10 gene expression. The R package of ‘limma’ was used to find the differential expression of genes between the high-NAT10 group and low-NAT10 group, named C2 (adjusted *P* < 0.05 and Log (Fold Change) >1 or Log (Fold change) <-1 was considered as the thresholds for the differential gene expressions). In total, 106 ac4C-related genes were obtained by using the intersection between C1 and C2. Heatmap and volcano plot were generated using the ‘ggplot2’ R package.

### Cox regression multivariable analysis and survival analysis

Multivariate Cox regression analysis was used to find the prognostic genes. Twelve differential genes were identified as risk genes, related to mRNA acetylation modification in colon cancer. *P* < 0.05 was considered statistically significant. The R package ‘survival’ was used for survival analysis. Survival data of 12 prognostic genes were analysed by using Kaplan-Meier curve. Genes *LGR5* and *CLDN9* were significantly correlated with survival (*P* < 0.05).

### Acetylation site prediction

LGR5 and CLDN9 mRNA sequences were obtained from the NCBI Gene database. Acetylated sites on RNAs were predicted by PACES [14].

### Construction of the ac4C risk score model

The RNA-seq data and clinical data of 106 ac4C-related differential genes were downloaded from TCGA-COAD. We applied the least absolute shrinkage and selection operator (LASSO) logistic regression algorithm for feature selection and 10-fold cross-validation was used. This was done using the R package ‘glmnet.’ The Kaplan-Meier survival analysis and Log-rank test were used to compare the survival differences between the high-risk and low-risk groups. Time-dependent receiver operator characteristic (ROC) curve analysis was utilized to assess the accuracy of the risk score for predicting survival using the ‘timeROC’ package. *P* < 0.05 was considered statistically significant.

### Gene Set Variation Analysis (GSVA) and GSEA

we performed GSVA enrichment analysis using ‘GSVA’ R packages to investigate the difference in biological process between the high and low ac4C risk score groups. Gene expression data of high and low ac4C risk score groups were prepared as input data for running GSVA analysis. The gene sets of ‘c2.cp.kegg.v7.2.symbols’ were downloaded from the MSigDB database. Adjusted *P* value<0.05 was considered statistically significant. The R package ‘pheatmap’ was used for creating the pathway heatmap.

Gene Set Enrichment Analysis (GSEA) is a computational method that determines whether a priori-defined set of genes shows statistically significant and concordant differences between two biological states. GSEA 4.1.0 software was used to analyse the differences between the NAT10 high expression and NAT10 low expression groups.

### Tumour mutation burden (TMB) analysis

Tumour mutation data were derived from The Cancer Genome Atlas (TCGA) (TCGA-COAD). Tumour mutation burden (TMB) was defined as the total number of non-synonymous mutations per coding area of a tumour genome [mutations per Megabase (Mb)], and UCSC hg38 was used as the reference genome. The mut number and quality of gene mutations were analysed by using R Maftools software package. TMB value was calculated by dividing the number of non-synonymous mutations by the total length of exon. For the specific method, the TMB value of each sample was calculated by TMB = mut number/38 (The total length of exon as 38 M). We compared the differences in TMB and mutational landscape between the high and low-risk groups. The mutation waterfall plot was drawn by R package ‘maftools.’

### The association between ac4Cscore and microsatellite instability (MSI) status in

*colorectal cancer*

MSI of colorectal cancer patients were was measured using the TCGA data portal. We classified the MSI status of colon cancer patients into MSI-high (MSI-H), MSI-low (MSI-L), or microsatellite stability status (MSS). The percentages of MSI-H, MSI-L, and MSS in the high and low ac4C risk score groups were calculated by R package ‘plyr.’ For differences in ac4C risk scores among the MSI-H, MSI-L, and MSS groups, boxplots were completed with the R package ‘ggplot2.’

### Prediction of cancer immunotherapy response

The prediction of colorectal cancer patients immunotherapy response data were obtained from the TIDE and TCIA databases. TIDE Score, Dysfunction Score, Exclusion Score, MDSC, CAF, TAM M2 and IPS Score were used to compare the response to immunotherapy between high and low ac4C risk score groups.

### Cell culture

HCT116, HT29, DLD1, and NCM460 cells were cultured in 1640 medium (Hyclone, USA) with 10% foetal bovine serum (ABW). SW480 cells were cultured in DMEM medium (Hyclone, USA) with 10% foetal bovine serum (ABW). All cells were obtained from China Center for Type Culture Collection (Wuhan, CN).

### Immunohistochemistry

In total, 24 colon cancer tissue samples and 24 normal colon tissue samples were obtained from Zhongnan Hospital of Wuhan University. The study was approved by the ethics committee of Zhongnan Hospital of Wuhan University (#2,020,150) and informed consent was acquired from each patient. Samples were embedded in paraffin and sectioned after dehydration and wax immersion. For immunohistochemistry, paraffin sections were dewaxed and underwent antigen retrieval with EDTA buffer. Then, sections were blocked with 1% foetal bovine serum (ABW) for 30 min at 37°C The sections were incubated overnight with NAT10 Rabbit mAb (ABclonal, A19286, Wuhan, CN) for staining. Next, sections were incubated with HRP-labelled anti-rabbit or anti-mouse secondary antibodies (Proteintech, PR30009, Wuhan, CN）for 2 h. After incubation with DAB (ZSGB-BIO, ZLI-9018, CN) for 1 min, photographs were taken using fluorescence microscopy (Olympus, Japan).

### RNA dot blot

The RNA was heated at 95°C for 2 min. RNA concentration was measured by Nanodrop 2000 (ThermoFisher, USA). Then, 5 ug of total RNA was added to the nitrocellulose filter membrane (Abcolne, CN) and dried at room temperature for 30 min, Thereafter, the sample was blocked with 5% BSA, and incubated with anti-N4-acetylcytidine (ac4C) antibody (Abcam, ab252215) overnight at 4°C. Anti-rabbit IgG-HRP (Servicebio, GB23303, CN) was used as the secondary antibody.

### Western blot

CRC cell lines HCT116, DLD1, HT29, and SW480 and the human colon epithelium NCM460 cells were lysed with NP40 lysis buffer (Beyotime, P0013F, CN). Proteins were separated on 10% SDS-polyacrylamide gel electrophoresis (SDS-PAGE) and transferred to a polyvinylidene fluoride (PVDF) membrane (Millipore, IPFL00010, GER). Then, the membrane was blocked with 5% skim milk for 1 h. The membrane was then incubated with primary antibodies for GAPDH (Proteintech, 10494–1-AP, Wuhan, CN), MLH1 (ZENBIO, R22726, CN), PMS2 (ZENBIO, R22726, CN), MSH2 (Proteintech, 15520–1-AP, CN), MSH6 (ZENBIO, R27040, CN) and NAT10 (ABclonal, A19286, Wuhan, CN) overnight at 4°C. Thereafter, the membrane was incubated with the secondary antibody anti-rabbit IgG-HRP (Servicebio, GB23303, CN, 1:5000 dilution) for 2 h at room temperature. Finally, the protein band was detected with using Omni-ECL™Pico Light Chemiluminescence Kit (epizyme, SQ202L, Shanghai, CN).

### Gene overexpression and knockdown

NAT10-overexpressing plasmid pECMV-NAT10-3×FLAG and matched empty vector were purchased from miaolingbio Company (Wuhan, CN). NAT10 siRNA was purchased from Tsingke Biotechnology. Cells were transfected with lipo8000 (Beyotime, C0533, CN).

### Cell proliferation assay

HT29 and HCT116 cells were seeded in a 96-well plate at a cell density of 1000 cells per well with 100 μL of media per well. Following incubation for 24 h, cells were transfected with pECMV-NAT10-3×FLAG or empty vector. After transfection, 10 µL of CCK-8 solution (Beyotime, Shanghai, China) was added to each well at 0, 24, 48, 72, and 96 h. After incubation for 2 h, absorbance was read at 450 nm using a microplate reader (BioTek, US).

### Statistical analysis

R version 4.0.5 and GraphPad Prism 8.0 were used to analyse data as mentioned above. Student’s t-test was used to measure statistical significance. Correlations between the two groups were evaluated using the Pearson test for parametric variables. *P* < 0.05 was considered significant.

## Results

### High expression of NAT10 in colorectal cancer

**Figure 1.**
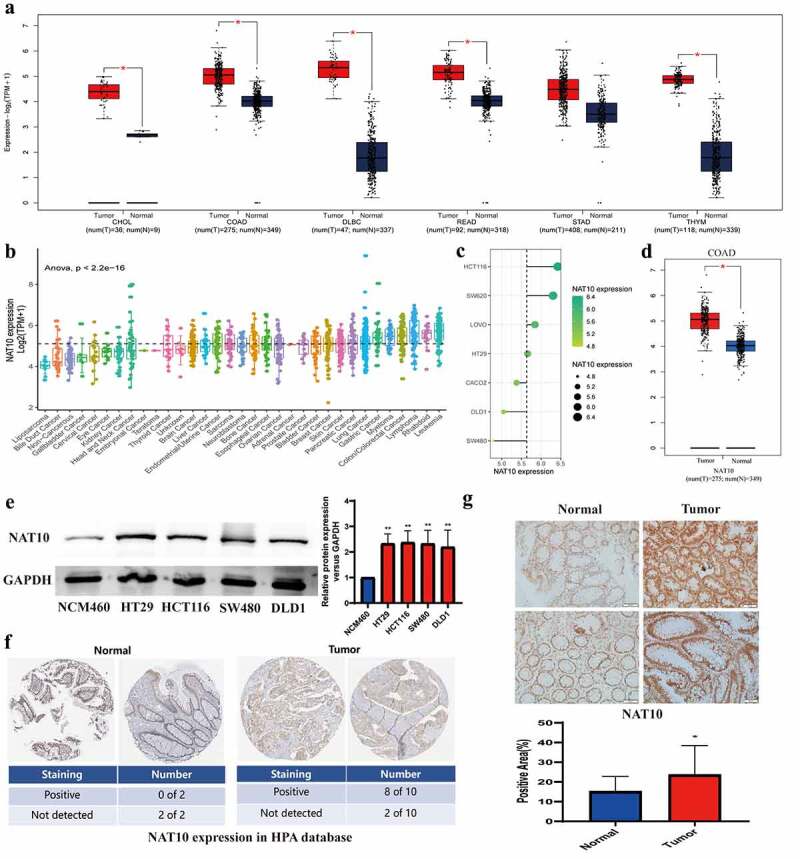
The expression of NAT10 in CRC and other tumours.(a, d) by using the GEPIA database, results showed NAT10 expression in different tumour tissue and matched normal tissue.(b, c) NAT10 expression levels in different tumour cell lines. Data were obtained from CCLE.(e) Western Blot detected the NAT10 expression between normal colon cancer epithelial cells (NCM460) and other colon cancer cells (HT29_`_ HCT116_`_ SW480 and DLD1), and GAPDH expression was used to calibrate differences between groups.(f) the expression data of NAT10 in pathological sections come from the human protein atlas.(g) the expression data of NAT10 in pathological by IHC and the tissues are from Zhongnan Hospital of Wuhan university.

First of all, we evaluated the expression of NAT10 in 31 different types of tumours using the TCGA and GTEx datasets. We found that NAT10 was overexpressed in many types of tumour tissues including gastric cancer, colon cancer, rectal cancer, B-cell lymphoma, and thymic carcinoma. (Figure 1a). We also analysed the expression levels of NAT10 in different types of cancer cell lines from the CCLE database. NAT10 had the highest expression in colon cancer, lymphoma, rhabdoid, and leukaemia (Figure 1(b,c and d)).

Next, we validated the high expression of NAT10 in colon cancer cell lines HT29, HCT116, SW480, and DLD1, compared with normal intestinal mucosal epithelial cell line NCM460 (Figure 1e). We also measured the expression level of NAT10 in the colorectal cancer tissue samples. According to the Human Protein Atlas database, 8 out of 10 colorectal cancer samples were positive for NAT10 in immunohistochemical staining, while normal colon samples were all negative for NAT10 expression (Figure 1f). We collected 24 normal and 24 colon cancer tissue specimens to verify the expression of NAT10 in colon cancer. Immunohistochemical staining indicated that NAT10 was highly expressed in colon cancer tissues (Figure 1g, Figure S1). Patient information can be obtained in Supplementary Table S1.

### Biological functions of NAT10 in colorectal cancer

**Figure 2.**
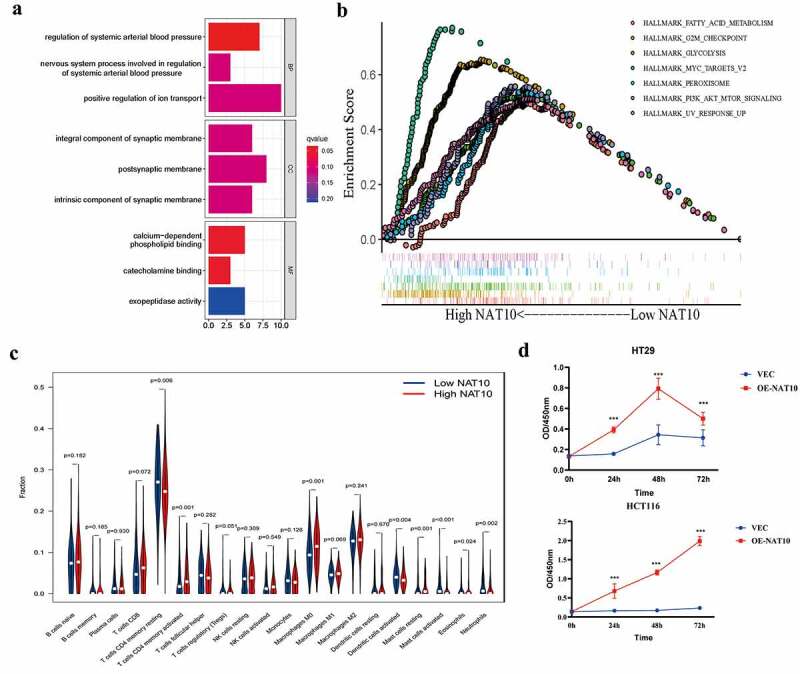
Biological functions of NAT10 in CRC.(a) KEGG analysis based on DEGs between High NAT10 and Low NAT10 CRC patients.(b) GSEA analysis between high and low expression of NAT10 gene group. ES means enrichment score. (c) Analysis of immune cell infiltration between high and low expression NAT10 groups by using the CIBERSORT algorithm. *P* < 0.05 is considered statistically significant.(d) CCK8 detected the proliferation ability of HCT116 and HT29 cells after NAT10 overexpression, and the data was the average value after repeated three times.

We investigated the biological function of NAT10 in colorectal cancer. NAT10-related signalling pathways were analysed using KEGG pathways and the differential genes obtained from the high and low groups. The results showed that NAT10 is involved in the positive regulation of ion transport, postsynaptic membrane, and calcium-dependent phospholipid binding (Figure 2a). Further GSEA analysis showed that RNA acetylation modification caused by NAT10 mainly affects fatty acid metabolism, G2M checkpoint, and MYC pathways (Figure 2b). Immune infiltration analysis showed that immune infiltration was relatively high in the high NAT10 expression group. In particular, activated CD4 T cells and macrophage M0 infiltration level was significantly higher in the high NAT10 expression group than in the low NAT10 expression group (Figure 2c)

In vitro experiments also showed that NAT10 can significantly affect colon cancer cell proliferation. CCK8 results showed that colon cancer cell proliferation was significantly enhanced after NAT10 overexpression (Figure 2d). These findings show that NAT10 is significantly involved in the development of colorectal cancer by increasing the level of RNA acetylation, immune invasion and colorectal cancer cell proliferation.

### Identification of NAT10-related ac4C-DEGs in colorectal cancer

**Figure 3.**
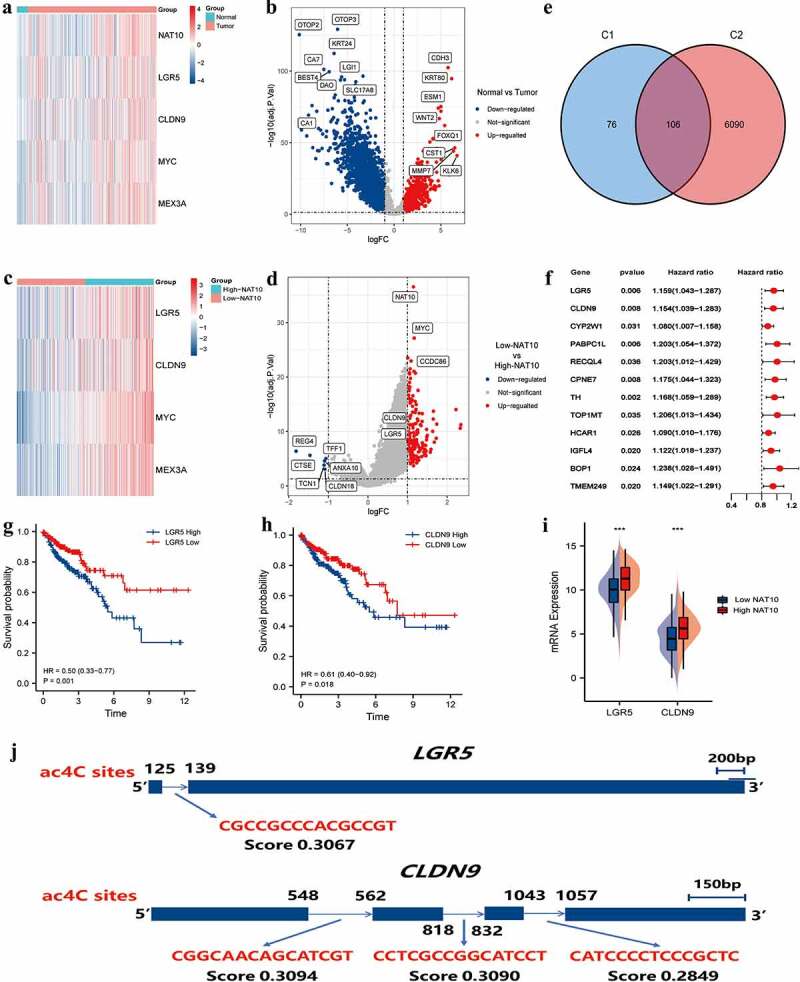
Identification of NAT10-related ac4C-DEGs in CRC. (a) the heatmap showed differential expression genes between normal and CRC tissues. |log(fold Change)| < 1 and *p* < 0.05 was considered as the thresholds for the differential gene expressions. We chose the expression of 5 genes (NAT10, LGR5, CLDN9, MYC, and MEX3A) to plot the heatmap. (b) Volcano plot showing the distribution of differential genes in the TCGA-COAD dataset between normal and CRC tissues. (c) the heatmap showed differential expression genes between high and low NAT10 groups. |log(fold Change)| < 1 and *p* < 0.05 was considered as the thresholds for the differential gene expressions. We chose the expression of 4 genes (LGR5, CLDN9, MYC, and MEX3A) to plot the heatmap. (d) Volcano plot showing the distribution of differential genes in the TCGA-COAD dataset between normal and CRC tissues. (e) the Venn diagram shows the intersection of genes between two groups C1 (DEGs of high and low NAT10 groups) and C2 (DEGs of normal and tumour) of differential genes.

We identified genes differentially expressed in tumour tissues and normal colon tissues (Figure 3(a and b)). NAT10 is the only confirmed regulator of mRNA acetylation and has multiple functions in cancer. However, the potential mechanisms by which NAT10 modulates tumour initiation and progression have not yet been elucidated. Therefore, we compared the DEGs between tumours with high and low expression of NAT10 (Figure 3(c and d)). After intersecting two groups of differential genes on the Venn diagram, 106 differential genes were obtained (Figure 3e). Survival analysis was performed with the 106 differential genes to find the genes that were most relevant to clinical prognosis. Among them, 12 genes were significantly associated with the prognosis of colorectal cancer (Figure 3f).

The high expression of *LGR5* and *CLDN9* were closely associated with poor prognosis in CRC patients (Figure 3(g and h)). Meanwhile, both *LGR5* and *CLDN9* were highly expressed in the high-NAT10 group (Figure 3i). Additionally, we predicted ac4C modification sites on *LGR5* and *CLDN9* through PACES (Figure 3j). The mRNA fragment of *CLDN9* had three possible acetylation sites, therefore, mRNA acetylation of *CLDN9* is likely to affect the gene function.

### Prognostic model based on NAT10-related ac4C-DEGs

**Figure 4.**
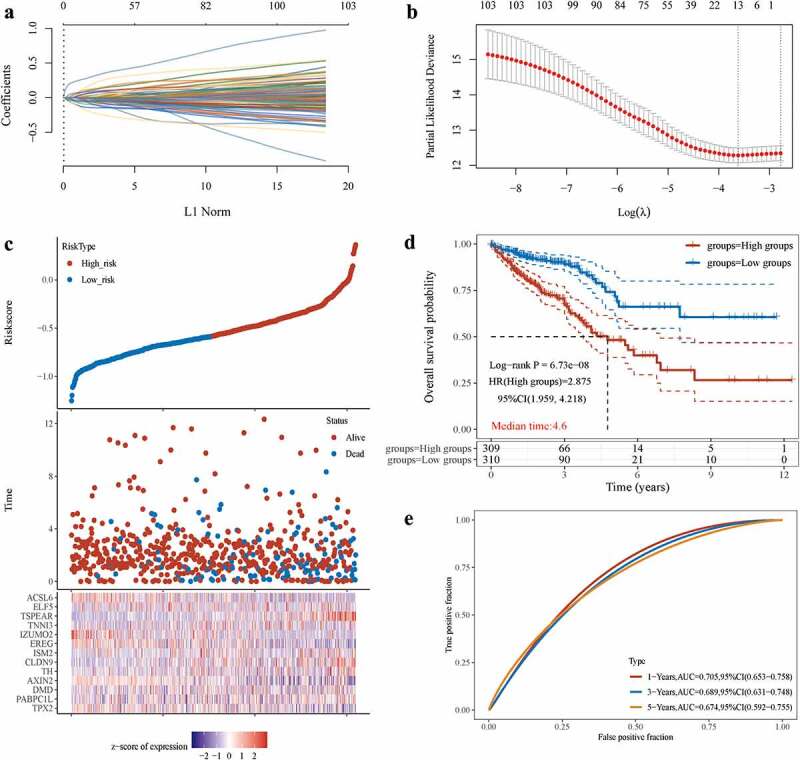
Prognostic model of NAT10.(a) LASSO coefficients profiles of 103 RNA acetylation genes. (b) LASSO regression with tenfold cross-validation obtained 13 prognostic genes using minimum lambda value. (c, d) Relationship between RNA acetylation risk score of colorectal cancer patients, survival analysis showed the OS between high-risk score group and low-risk score group, Log-rank *P* < 0.001.(e) the AUC curve was used to evaluate the model’s predictive ability. The higher the AUC result, the stronger the predictive ability, 1-Years AUC = 0.705.

We constructed a scoring acetylation model to assess the clinical relevance of RNA acetylation. To avoid the overfitting phenomenon in the subsequent model construction, we used a least absolute shrinkage and selection operator (LASSO) regression to detect whether dimensionality reduction is possible by eliminating redundant genes. According to the partial likelihood deviance and lambda values, 13 genes were included in the model construction (Figure 4(a and b)). The lasso risk score was obtained by the following formula: Riskscore=(0.0446)*TPX2+(0.0338)*PABPC1L+(0.0387)*DMD+(−0.0868)*AXIN2+(0.0484)*TH+(0.0602)*CLDN9+(0.0034)*ISM2+(−0.0287)*EREG+(−0.089)*IZUMO2+(0.0054)*TNNI3+(0.2255)*TSPEAR+(0.0034)*ELF5+(−0.0485)*ACSL6.

The lasso regression provided a risk score of mRNA acetylation for each sample based on the expression level of the included genes. The samples were divided into two groups in a dichotomous fashion based on their risk score ranking (Figure 4c). Patients with low lasso risk scores had longer survival compared with patients with high lasso risk scores (Figure 4d). We generated a receiver operating characteristic (ROC) curve to assess the predictive accuracy of this model (Figure 4e). The above analysis suggests that mRNA acetylation risk score is positively correlated with clinical outcomes.

### Characteristics of the tumour microenvironment in different ac4Cscore groups

**Figure 5.**
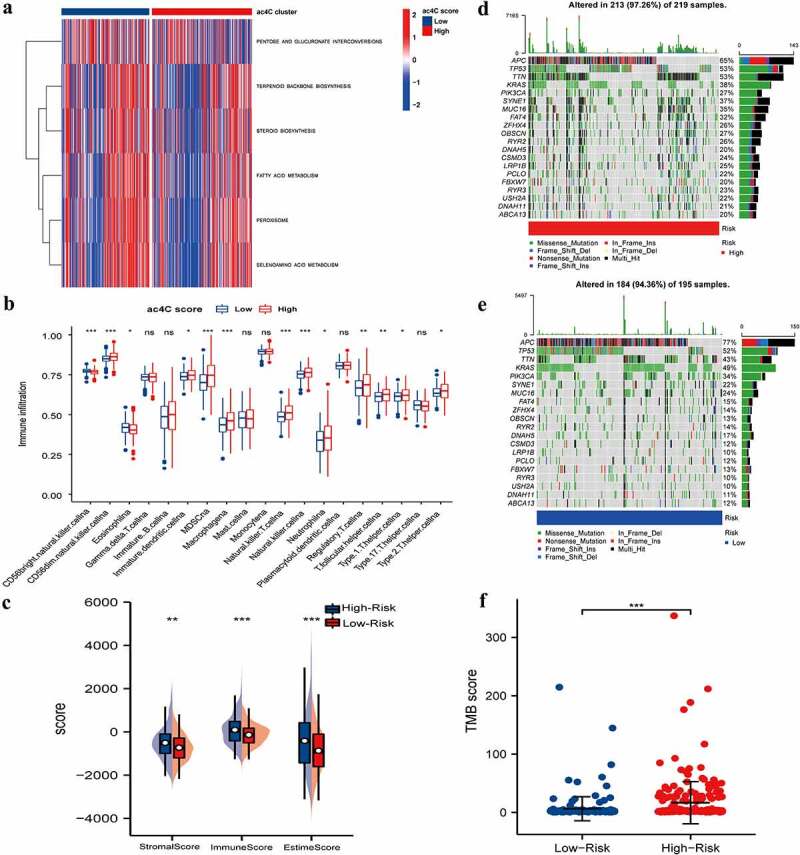
Tumour Microenvironment of ac4cscore Groups.(A) Pathway differences between high and low acetylation risk score groups using GSVA analysis, adj.P.value<0.05 is considered statistically significant.(b, C) ssGSEA Analysis of Differences in Immune Cell Infiltration Levels and low-risk groups. CIBERSORT was used to calculate and compare stromal scores, immune scores, and estimate scores in the high and low acetylation risk score groups.(d, E) the waterfall plot depicts SNV and INDL in patients in the acetylation risk score group(f) Compare TMB scores between high and low-risk groups, TMB is calculated as ‘number of somatic mutations/length of corresponding region.

We compared different gene pathways between high and low mRNA acetylation risk score groups using GSVA to investigate the relationship between mRNA acetylation risk score and tumour immune infiltration. Low mRNA acetylation risk score was related to peroxisome, selenoamino acid metabolism, and fatty acid metabolism pathways (Figure 5a). Furthermore, we compared the genetic and immune characteristics of patients in different ac4Cscore groups. We compared tumour immune infiltration between the two groups. Tumour immune infiltration was higher in the high-risk score group than that in the low-risk score group. Most types of immune cells, such as MDSC, NK cells, and NK-T cells, were more abundant in the high ac4C score group (Figure 5b).

We also evaluated the difference between the two groups in terms of the stromal score, immune score, and estime score. Similar to immune infiltration, the high-risk group had higher stromal, immune, and estime scores (Figure 5c). We depicted the somatic mutation landscapes in the high and low ac4Cscore groups. Mutation frequency was high in the high-risk group and relatively low in the low acetylation risk score group (Figure 5(d and e)). Additionally, the TMB score was calculated and the Wilcoxon test showed that the TMB scores were significantly higher in the high-risk group than in low-risk group (Figure 5f). These findings suggest that high acetylation risk scores are correlated with tumour immune microenvironment and tumour mutation burden.

### Association between ac4Cscore and MSI status in colorectal cancer

**Figure 6.**
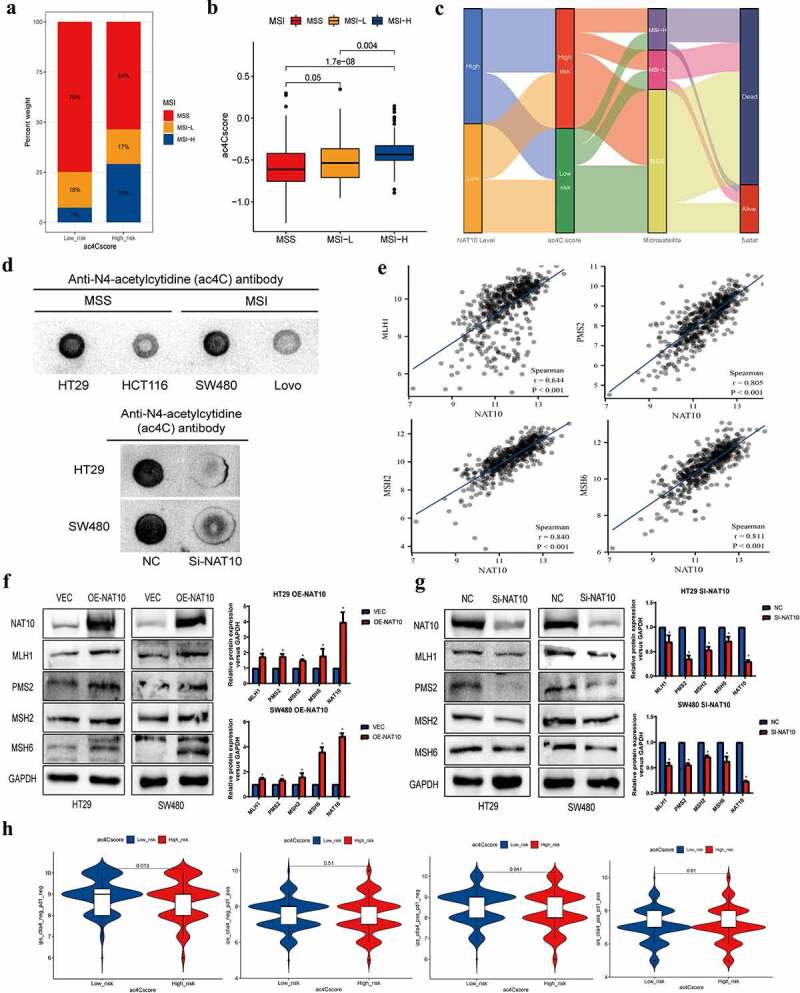
Ac4cscore and microsatellite instable (MSI) status in CRC.(a, b) the vertical stack bar gram showed the proportions of MSS_`_ MSI-L and MSI-H in the high and low acetylation risk groups. Grouping histogram showing the differences in mRNA acetylation risk scores between different microsatellite statuses.(c) Alluvial diagram depicts the attribution of microsatellite status subtypes in the ac4cscore groups.(d) Detection of RNA acetylation level using RNA dot blot in MSS cell lines HT29, SW480 and MSI cell lines HCT116, Lovo. The total quality of RNA added to each spot is 5ug.(e) Correlation analysis reveals the association between NAT10 and MLH1_`_ MSH2_`_ PMS2 and MSH6.(f, g) NAT10 was overexpressed and knocked down in HT29 and SW480 cell lines respectively, and the expression of four mismatch repair proteins MLH1, PMS2, MSH2, MSH6 was detected by western blot.(h) Relationship between mRNA acetylation risk score and therapeutic response to PD-1 and CTLA4, the treatment response data from TCIA.

Recent studies have shown that the microsatellite status of colon cancer can affect the level of tumour immune invasion [15]. Therefore, we further investigated the effect of mRNA acetylation risk score on microsatellite status in colon cancer. We evaluated the difference in ac4Cscore between MSI and MSS in colon cancer. Higher ac4Cscore was associated with MSI subtype, while lower ac4Cscore was associated with the MSS subtype (Figure 6a). The high microsatellite instability (MSI-H) subtype was significantly correlated with higher ac4Cscore, whereas the low microsatellite instability (MSI-L) subtype and MSS were significantly correlated with lower ac4Cscore (Figure 6b). Consistent with the above findings, the alluvial diagram showed that the ac4Cscore high-risk group mainly overlapped with the NAT10 high-level group, and the high ac4Cscore group mainly overlapped with the MSI subtype (Figure 6c).

We further compared the acetylation levels between MSS colon cancer cell lines (HT29 and SW480) and MSI colon cancer cell lines (HCT116 and Lovo). The results showed that RNA acetylation level was significantly higher in MSS colon cancer than in MSI colon cancer (Figure 6d). After NAT10 knockdown in MSS cell lines, RNA acetylation level was significantly reduced. Mutations or deletions of mismatch repair genes such as MLH1, MSH2, PMS2, and MSH6 are common in MSI colorectal cancer [16]. Therefore, we analysed the relationship between the expression of NAT10 and four mismatch repair proteins in colorectal cancer patients. The results showed that the expression of NAT10 was positively correlated with the expression of MLH1, MSH2, PMS2, and MSH6 (Figure 6e). We also detected the expression levels of the mismatch repair proteins after overexpression and knockdown of NAT10 in colon cancer cell lines. The mismatch repair proteins, including MLH1, MSH2, PMS2, and MSH6, were upregulated after NAT10 overexpression, while downregulated after NAT10 knockdown (Figure 6(f and g)).

In current immunotherapy, colon cancer with MSI-H is sensitive toICIs [17]. Given the association between MSI and ac4Cscore, we investigated whether ac4Cscore predicts could indicate patient response to ICIs (Figure 6h).

## Discussion

The role of RNA modification in colorectal cancer has increasingly received attention in recent years [18,19]. RNA acetylation modification is a conservative type of chemical modification in human and yeast cells [20]. Previous studies have shown that mRNA acetylation can improve mRNA stability and enhance translation [10]. In an earlier study, N4-acetylcytidine (ac4C) levels were significantly elevated in urine samples of patients with rectal cancer [21]. However, our understanding of mRNA acetylation in colorectal cancer is still in its infancy due to the limited detection of early nucleic acid modification sites [22]. Therefore, this study used some in vitro experiments and bioinformatics to explore the role of mRNA acetylation in colorectal cancer.

N-acetyltransferase 10 (NAT10) encodes RNA cytidine acetyltransferase and is the only enzyme involved in RNA acetylation modification. It catalyzes mRNA ac4C modification [10,23]. NAT10 also catalyzes rRNA and tRNA ac4C modification [24,25]. Our study showed that NAT10 is highly expressed in human colon cancer tissues. The results of KEGG and GSEA functional and pathway analysis indicated that NAT10 is associated with ion transport, calcium-dependent phospholipid binding and RNA stability. In addition, we found that NAT10 is related to tumour immune infiltration. The CIBERSORT algorithm indicated that NAT10 is positively correlated with CD4+ T cells activation and negatively correlated with CD4+ T cells inactivation. High expression of NAT10 was also positively correlated with M0 macrophages infiltration. Exposure to foreign antigens activated CD4 + T cells. CD4 + T cells play a central role in the immune system and can rapidly release a large amount of cytokines [26,27]. M0 macrophages are quiescent macrophages that can differentiate into M1 (pro-inflammatory) or M2 (anti-inflammatory) types in tumour microenvironments. They are involved in immune regulation, inflammatory responses, tumour proliferation, and apoptosis [28–30]. NAT10 is involved in the development of colorectal cancer possibly by regulating immune infiltration.

Next, we divided colon cancer patients into the NAT10 high expression and NAT10 low expression groups. Using the intersection of differential genes between the NAT10 high expression and NAT10 low expression groups and between colon cancer tissue and normal tissue, we identified 106 acetylation- related differential genes. Next, we identified genes with the highest correlation with patient prognosis through LASSO regression analysis. We also constructed an acetylation-related risk score model. The results of GSVA analysis showed that the peroxisome, selenoamino acid metabolism and fatty acid metabolism pathways were inhibited in the high-risk score group. There was a significant difference in survival between the high-risk score and low-risk score groups. The high-risk score group was associated with poor prognosis in colorectal cancer patients.

Tumour immunotherapy has become the main treatment for colorectal cancer, but unfortunately, only a few patients with MSI colorectal cancers effectively respond well to immunotherapy [31,32]. MSS colorectal cancer has an extremely low clinical response rate to programmed cell death protein 1 (PD-1) antibodies [32]. Tumour immune infiltration is also significantly reduced in MSS-type colorectal cancer. In addition, impaired immune infiltration in MSS-type colorectal cancer is less responsive to drugs such as ICIs, known as ‘cold tumour’ [33].

The relationship between RNA acetylation levels and colorectal cancer microsatellite stability, and response to immunotherapy is unclear. Therefore, we investigated this relationship. The results showed that NAT10 expression was strongly correlated with the expression of the four mismatch repair proteins. In patients with high acetylation scores, the proportion of MSI-type colorectal cancer increased. Consistently, the acetylation risk score was higher in MSI-type colorectal cancer patients than in MSS-type colorectal cancer patients. TMB and immune cell infiltration were higher in the hyperacetylation group than in the hypoacetylation score group. We further predicted responses to immunotherapy in patients with high risk scores and found that RNA acetylation scores were associated with response to immunotherapy. In conclusion, mRNA acetylation is related to tumour immune infiltration in colorectal cancer and improves tumour response to immunotherapy. Therefore, mRNA acetylation-related genes are expected to promote the transformation of ‘cold’ tumours to ‘hot’ tumours.

In conclusion, we verified that NAT10 plays an important role in colon cancer using databases and in vitro experiments. NAT10 is highly expressed in colon cancer tissues and promotes colon cancer proliferation. Based on mRNA acetylation-related differential genes in colon cancer, we developed an acetylation risk score model. It showed that high RNA acetylation risk scores were associated with tumour immune infiltration levels, TMB, and microsatellite status. Patients in the high-risk group had significantly worse prognoses than those in the low-risk group. This suggests that mRNA acetylation may play an important role in colon cancer microsatellite status and response to immunotherapy. Using NAT10 as a target to develop clinical drugs has strong potential clinical value However, the limitation of this study is that further studies are still needed to comprehensively explore the key genes and specific modification sites of RNA acetylation modification, as well as the specific mechanism o by which RNA acetylation affects the response to immunotherapy in colorectal cancer.

## Supplementary Material

Supplemental Material
