# Supplementary material for: NAT10 mediated mRNA acetylation modification patterns associated with colon cancer progression and microsatellite status
Source: Epigenetics. 2023 Mar 12;18(1):2188667. doi: 10.1080/15592294.2023.2188667 (PMC10026876; doi:10.1080/15592294.2023.2188667)

Table S1. The characteristics of colorectal cancer patients

| Number | Gender | Age | Pathologic | TNM |
| --- | --- | --- | --- | --- |
| 44 | female | 45 | carcinoma of sigmoid | T3N0Mx |
| 49 | male | 61 | rectal carcinoma | T3N2aMx |
| 69 | female | 65 | colon carcinoma | T4aN1cM1 |
| 70 | male | 73 | carcinoma of sigmoid | T3N1bMx |
| 90 | female | 54 | colon carcinoma | T1N0Mx |
| 95 | male | 46 | colon carcinoma | T3N0Mx |
| 104 | male | 63 | colon carcinoma | T2N0Mx |
| 114 | female | 61 | colon carcinoma | T3N0Mx |
| 118 | male | 58 | colon carcinoma | T1N0Mx |
| 121 | male | 52 | colon carcinoma | T4aN1cM1 |
| 141 | male | 57 | colon carcinoma | T2N0Mx |
| 147 | male | 81 | colon carcinoma | T3N0Mx |
| 149 | male | 31 | rectal carcinoma | T3N0Mx |
| 152 | male | 66 | colon carcinoma | T1N0Mx |
| 167 | female | 67 | colon carcinoma | T2N0Mx |
| 175 | female | 55 | rectal carcinoma | T3N0Mx |
| 176 | male | 74 | colon carcinoma | T3N0Mx |
| 190 | male | 75 | rectal carcinoma | T1N0Mx |
| 191 | male | 52 | colon carcinoma | T3N0Mx |
| 194 | male | 81 | colon carcinoma | T1N0Mx |
| 196 | male | 72 | rectal carcinoma | T3N0Mx |
| 197 | male | 72 | rectal carcinoma | T3N0Mx |
| 204 | female | 72 | colon carcinoma | T2N0Mx |
| 205 | male | 74 | rectal carcinoma | T3N1bMx |

Figure legend

Figure S1. Immunohistochemical of NAT10 in 24 patients with colorectal cancer

Figure S1


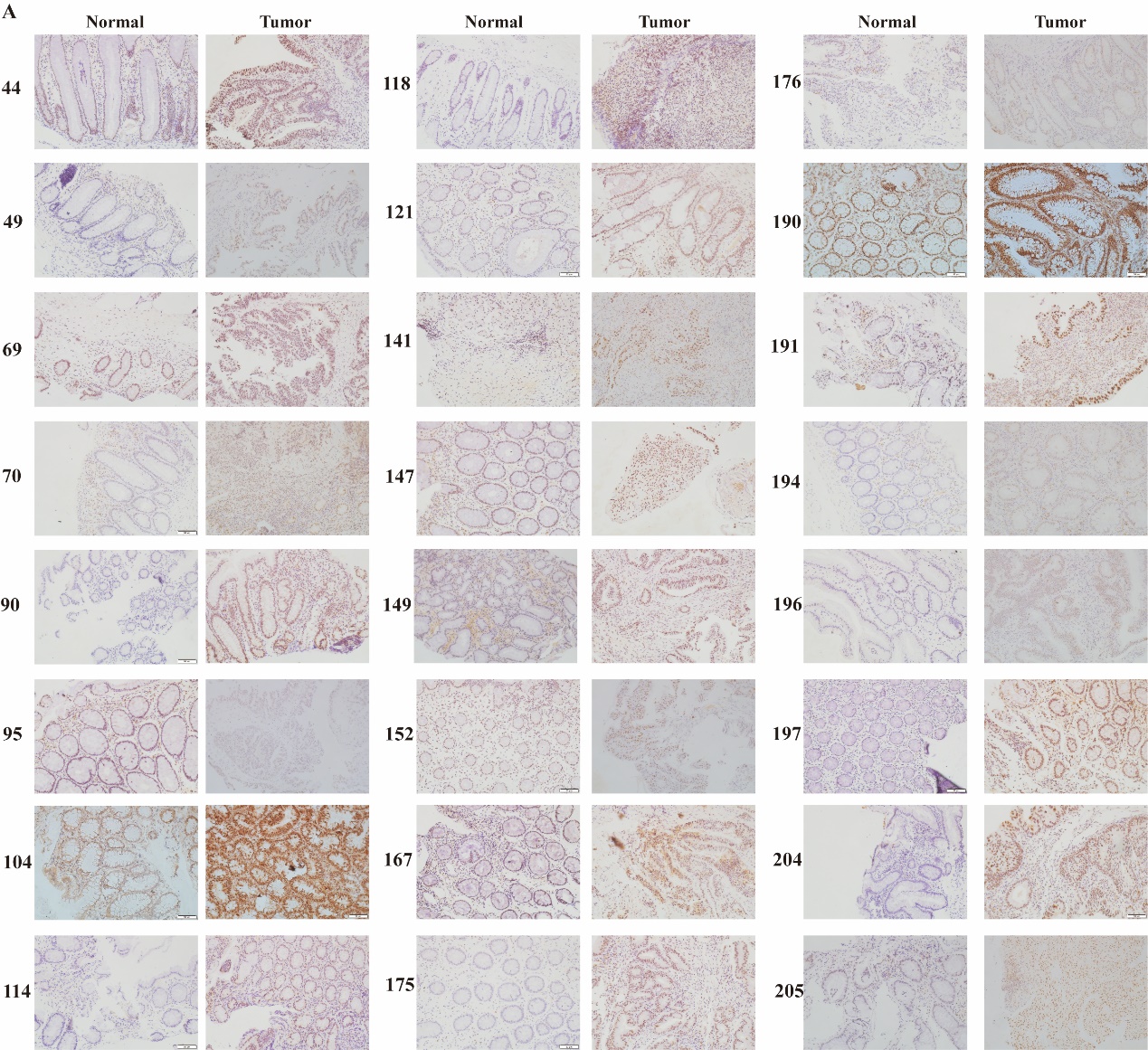

Supplement: Supplemental Material [file KEPI_A_2188667_SM0244.zip › Supplementary files/supplymentary.docx]
