# Supplementary figures and images for: NAT10 mediated mRNA acetylation modification patterns associated with colon cancer progression and microsatellite status
Source: Epigenetics. 2023 Mar 12;18(1):2188667. doi: 10.1080/15592294.2023.2188667 (PMC10026876; doi:10.1080/15592294.2023.2188667)

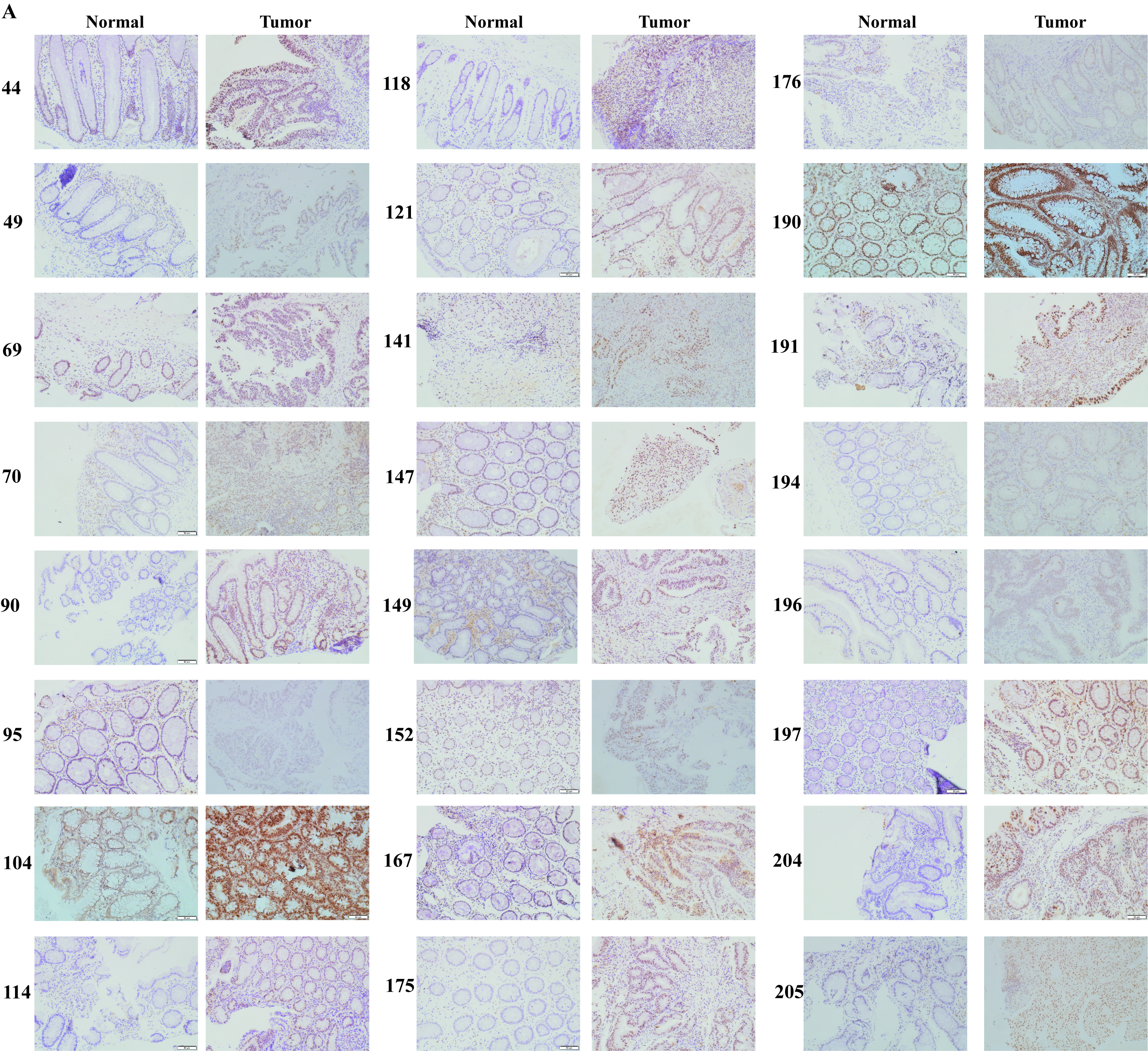

Supplement: Supplemental Material [file KEPI_A_2188667_SM0244.zip › Supplementary files/FIG S1.tif]
